# The Mediating Role of Inner Strength in the Relationship Between Illness Perception and Self-Management in Older Adults with Advanced Lung Cancer: A Cross-Sectional Study

**DOI:** 10.3390/healthcare14101257

**Published:** 2026-05-07

**Authors:** Fangjia Shen, Yinlou Ling, Caixia Ke, Jing Chen, Xinyi Li, Mingfang Li, Yantong Xie, Zhiqi Yang, Siyu Guan, Yaqian Huang, Yuqi Qiu, Jie Mei, Yueli Wang, Jun Yan

**Affiliations:** 1 School of Nursing, Sun Yat-sen University, Guangzhou 510000, China; 2 Nursing Department, The Second Hospital Affiliated to Zhejiang University, Hangzhou 310000, China; 3 The First Hospital Affiliated to Sun Yat-sen University, Guangzhou 510000, China; 4 School of Nursing, Li Ka Shing Faculty of Medicine, University of Hong Kong, Hong Kong SAR, China; 5 Zhongshan Ophthalmic Center, Sun Yat-sen University, Guangzhou 510000, China; 6 Sun Yat-sen University Cancer Center, Guangzhou 510000, China

**Keywords:** older adults, advanced lung cancer, self-management, illness perception, inner strength

## Abstract

**Highlights:**

**What are the main findings?**

- Self-management of older adults with advanced lung cancer remains suboptimal.
- Inner strength partially mediates the association between illness perception and self-management.

**What are the implications of the main findings?**

- This study suggests that inner strength partially mediates the association between illness perception and self-management, extending the Stress and Coping Theory by identifying it as a mobilizable buffer against negative disease cognitions.
- The findings demonstrate that positive illness perception may promote self-management via inner strength, providing a potential evidence base for developing psychological interventions that empower older adults to cope with advanced lung cancer.

**Abstract:**

**Objectives**: This study aimed to investigate the associations between inner strength (IS), illness perception (IP) and self-management (SM) in older adults with advanced lung cancer. **Methods**: A cross-sectional survey was conducted with 222 patients recruited via convenience sampling between December 2021 and February 2023. SM, IP, and IS were measured using the Cancer Self-Management Assessment Scale, the Revised Illness Perception Questionnaire for lung cancer patients, and the Inner Strength Scale. Data were analyzed using descriptive statistics, Pearson correlation analysis, and structural equation modeling (SEM). **Results**: Participants exhibited moderate levels of SM, with the lowest scores observed for the symptom management dimension. Both SM and IS were negatively associated with negative illness perception (NIP) (*r_SM_*: −0.500, −0.149, *p* < 0.05; *r_IS_*: −0.519, −0.250, *p* < 0.001), and positively associated with positive illness perception (PIP) (*r_SM_*: 0.360, 0.565, *p* < 0.001; *r_IS_*: 0.233, 0.467, *p* < 0.001). Among the various dimensions of IP, personal control (representing PIP) and emotional representations (representing NIP) exhibited the strongest associations with SM (*r_PIP_* = 0.565, *p* < 0.001; *r_NIP_* = −0.500, *p* < 0.001). A positive relationship was observed between SM and IS (*r* = 0.427, *p* < 0.001). SEM indicated that IS partially mediated the associations between SM and both NIP and PIP (*β_NIP_* = −0.120, 95%CI_NIP_: −0.234, −0.013, *p* = 0.032; *β_PIP_* = 0.132, 95%CI_PIP_: 0.011, 0.246, *p* = 0.028). **Conclusions**: The SM of older adults with advanced lung cancer remains suboptimal. IS may play an important mediating role in the association of both NIP and PIP with SM. These findings highlight that both IP and IS are key factors associated with SM in older adults with advanced lung cancer. Therefore, future research and clinical interventions should target these factors to improve SM in this population.

## 1. Introduction

Globally, lung cancer ranks first among all malignancies in both incidence and mortality, contributing to approximately 2.5 million new cases and over 1.8 million deaths per year [1]. Moreover, 70% of lung cancer cases occur in older adults (≥65 years), with approximately 75% of patients diagnosed at an advanced stage [2]. Within China’s rapidly aging population, the proportion of newly diagnosed advanced lung cancer cases among older adults is increasing [3]. Advances in medical treatment have increased the 5-year survival rate of patients with lung cancer to nearly 33% worldwide [4]. However, for patients who survive longer, disease management becomes more complex [5] owing to comorbidities, physical frailty, and declining organ function [6]. Patients with advanced lung cancer continue to face severe physical and emotional burdens, including psychological distress, persistent fatigue, cough, and dyspnea [7,8,9,10]. Effective self-management (SM) is essential to optimize health outcomes among older adults with advanced lung cancer [11,12,13].

SM refers to health behaviors adopted by patients to promote health outcomes and enhance quality of life (QoL) [14]. Prior empirical evidence demonstrates that effective SM significantly improves QoL and enables patients to sustain a crucial sense of control when facing advanced cancer [11,12]. However, despite its clinical benefits, SM is often suboptimal in older adults with advanced lung cancer, as their high SM demands are mismatched with limited SM behaviors. Key deficits typically include inadequate nutritional and exercise management, poor emotional coping strategies, barriers in the communication between patient and physician, and difficulty understanding complex medical information [15,16,17,18,19,20,21,22]. Consequently, merely recognizing the importance of SM is insufficient. There is an urgent need to explore the underlying mechanisms that influence SM in this vulnerable population to facilitate the development of effective intervention strategies. Significant predictors of SM may include illness perception (IP) and inner strength (IS) [23,24].

As a widely recognized factor that influences SM, IP refers to individuals’ cognitive and emotional responses to their disease, which are analytically grouped into two categories: negative IP (NIP) and positive IP (PIP) [23]. NIP reflects the perceived threat of the illness, encompassing dimensions such as timeline acute/chronic, timeline cyclical, consequence and emotional representations, which together reflect the perceived severity and emotional burden of the disease [25,26,27,28]. Conversely, PIP captures the positive aspects of IP related to the perceived controllability of the threat, including personal control, treatment control, and illness coherence [25,26,27,28]. Previous studies have explored the relationship between IP and SM in chronic disease populations [29,30,31]. Furthermore, with the shift in cancer care toward a chronic disease management model, the association between IP and SM has been extensively validated across general cancer populations [32,33,34]. Empirical studies of patients with lung cancer further support this perspective. Specifically, Kong et al. [35] suggested that higher NIP is correlated with poorer SM. Similarly, Browning et al. [36] observed that low PIP, reflecting insufficient confidence in personal and treatment control, was associated with a greater likelihood of engaging in harmful behaviors, such as continued smoking. Furthermore, Valentine et al. [9] found that patients with advanced lung cancer who perceived their condition with a “struggling” profile (implying high NIP) demonstrated the poorest psychological and physiological outcomes. Despite the well-established significance of IP, no research has specifically targeted older adults with advanced lung cancer. Given the unique physiological decline and cognitive characteristics of this group, the relationship between their IP and SM may differ from that observed in other groups and deserves investigation.

Beyond IP, IS is increasingly recognized as a key influencing factors of SM [24]. Specifically, IS is defined as a core psychological resource that is mobilized in the face of adversity, enabling individuals to affirm their self-worth, proactively embrace challenges, maintain connectedness with their environment, and reconstruct meaning in life [24]. Clarifying the distinction between IS and related concepts is essential to enhance the theoretical rigor of our conceptual framework. While resilience primarily denotes the capacity to recover from adversity, and self-efficacy refers to individuals’ beliefs in their ability to perform specific tasks, IS serves as a more integrative psychological resource [24,37,38]. For older adults with advanced lung cancer, IS may be particularly critical for sustaining SM. Beyond providing the psychological strength required to endure adversity, it may facilitate a transformative process whereby patients reconstruct life meaning by solving problems creatively and fostering profound environmental connectedness. This distinction highlights the unique role of IS within the present study.

Grounded in Stress and Coping Theory [39], individuals regulate stress through primary appraisal and secondary appraisal, the former involving the evaluation of the threat posed by a stressor and the latter concerning the assessment of available coping resources. In the context of advanced lung cancer, patients’ IP may reflect their primary appraisal of the disease threat, whereas IS may be understood as an important psychological resource mobilized during secondary appraisal. As a mobilizable psychological buffer, IS may help transform threatening illness-related cognitions into more positive SM behaviors, thereby providing a theoretical basis for its mediating role in the relationship between IP and SM. This role may be particularly vital among older adults with advanced lung cancer. Given the disease’s irreversible nature, the meaning-making process facilitated by IS may be essential for sustaining patients’ motivation to pursue SM goals, such as maintaining autonomy and QoL [40,41]. Existing studies provide indirect support for this proposed relationship [42,43]. Notably, Xiao et al. [44] showed that among lung cancer patients receiving chemotherapy, a group predominantly comprising older adults with advanced disease, higher levels of IS were associated with positive coping strategies, including proactively seeking information from healthcare providers and attempting to address difficulties in symptom management. Moreover, in older populations, IS has been shown to be associated with proactive SM behaviors, including shared decision-making and active information seeking [43]. In summary, besides helping older adults with advanced lung cancer to psychologically cope with the disease, IS may represent a potential pathway through which IP influences SM. However, no study to date has empirically tested this mediation mechanism in this population.

**Figure 1:**
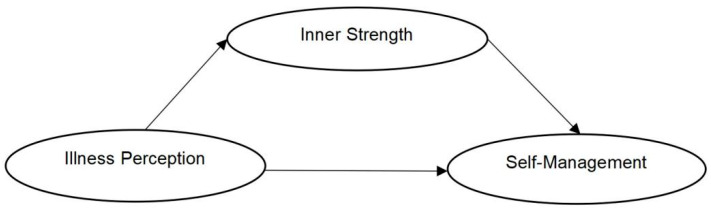
Conceptual model of the study.

To address this research gap, the present study examined the relationships among IP, IS, and SM in older adults with advanced lung cancer. Based on the theoretical framework and existing empirical evidence, this study hypothesized that IS would mediate the association between IP and SM (Figure 1).

## 2. Materials and Methods

### 2.1. Study Design, Setting, and Participants

The study used a cross-sectional design. Its reporting adheres to the STROBE guidelines. Eligible individuals were enrolled via convenience sampling from a large tertiary general hospital affiliated with Sun Yat-sen University in Guangzhou, China, between December 2021 and February 2023. The inclusion criteria were: (1) diagnosis of Stage III or IV primary lung cancer, (2) ≥60 years old, (3) aware of the cancer diagnosis, and (4) able to read and write in Chinese. The exclusion criteria were: (1) presence of cognitive impairment, (2) history of severe psychiatric illness, (3) presence of other malignant tumors, and (4) performance status (PS) ≥ 3. PS was assessed using the Eastern Cooperative Oncology Group (ECOG) scale, which ranges from 0 (fully active) to 5 (deceased) [45]. ECOG scores were extracted from medical records based on routine assessments conducted by attending physicians prior to enrollment. Patients with a PS ≥ 3 were excluded because impaired functional status may limit their ability to perform self-management [45].

### 2.2. Sample Size

The required number of participants was determined using a priori power analysis for the mediation model in G*Power 3.1.9.7 [46]. Based on an alpha of 0.05, power of 0.80, conservative effect size of 0.05, and two predictor variables (IS and IP), the minimum required sample size was computed as 196. To account for a potential 10% rate of invalid responses, the target sample size was increased to 218 participants. Ultimately, 222 valid questionnaires were collected, sufficient to meet the statistical power requirements.

### 2.3. Ethical Statement

The study was conducted in strict adherence to the tenets of the Declaration of Helsinki and received approval from the Research Ethics Committee of the Six Affiliated Hospital of Sun Yat-sen University (L2019ZSLYEC-0001). Prior to data collection, written informed consent was acquired from all participants.

### 2.4. Instruments

#### 2.4.1. Demographic and Clinical Data

Clinical and sociodemographic variables were obtained through a questionnaire designed by the researchers. The collected data included age, sex, educational level, per capita monthly household income, histological classification, tumour staging, disease duration, and performance status.

#### 2.4.2. Self-Management

The Cancer Self-Management Assessment Scale (CSMAS), developed by Cheng and Sun [14], was used to evaluate patients’ SM. The CSMAS comprises 44 items across six subscales: daily life management (11 items), symptom management (7 items), emotion management (9 items), communication with healthcare providers (4 items), information management (3 items), and self-efficacy (10 items). Each item is scored on a 5-point Likert scale ranging from 1 (never) to 5 (always), with higher scores suggesting better SM. As this study focused on behaviors rather than beliefs, the self-efficacy subscale was excluded, consistent with previous studies that adopted a behavior-focused approach to SM assessment [30,47]. In this study, the modified 34-item scale exhibited good reliability, with an overall Cronbach’s alpha of 0.92, and the subscale coefficients ranging from 0.72 to 0.93.

#### 2.4.3. Illness Perception

The Revised Illness Perception Questionnaire (IPQ-R, lung cancer) was used to measure participants’ IP [48]. Following recommendations of the IPQ-R’s authors, the causal and identity dimensions were revised to align with the specific characteristics of lung cancer [48]. The revision process comprised three stages. In Stage I, an initial version of the IPQ-R (lung cancer) was constructed based on a comprehensive literature review, resulting in a 19-item identity subscale and a 25-item causal subscale. In Stage II, six experts with specialized knowledge in respiratory nursing adjusted the subscales to ensure clarity and comprehensiveness. The initial subscales were thereby revised to a 13-item identity subscale and a 25-item causal subscale. In Stage III, nine experts with specialized knowledge in respiratory care or nursing evaluated the scales’ content validity. All of the subscales’ item-level content validity indices exceeded 0.78, while the scale-level content validity indices were 0.91 and 0.90 for the identity and causal subscales, respectively, indicating high content validity. The Cronbach’s alpha of these subscales ranged from 0.70 to 0.84. The finalized IPQ-R (lung cancer) comprises four sections: a 13-item identity subscale, a 25-item causal subscale, a 4-item subscale measuring NIP (timeline cyclical, timeline acute/chronic, consequence, and emotional representations), and a 3-item subscale measuring PIP (personal control, treatment control, and coherence) [26,27,28]. Items in the identity subscale were scored on a binary scale (1/0), while items in the other three subscales were scored on a 5-point Likert scale.

#### 2.4.4. Inner Strength

The revised Chinese version of the Inner Strength Scale (ISS) was used to assess participants’ IS [49]. The ISS comprises five dimensions: firmness (4 items), reflecting the courage to deal with stressful events; creativity (3 items), representing the proactive orientation to face, acknowledge, adapt to, and solve problems; courage (3 items), indicating the internal drive to face challenges and seize opportunities; connectedness (5 items), encompassing the internal drive to maintain friendships, communicate effectively, experience a sense of communion, and achieve transcendence; and flexibility (5 items), denoting the internal drive to endure and remain resilient when facing adversity [24]. All items are rated on a 6-point Likert scale ranging from 1 (strongly disagree) to 6 (strongly agree), with higher scores indicating higher levels of IS. In our study, the overall Cronbach’s alpha was 0.83.

### 2.5. Data Collection

Data were collected via convenience sampling in the departments of respiratory and oncology medicine at a large tertiary general hospital affiliated with Sun Yat-sen University in Guangzhou, China, between December 2021 and February 2023. The data collection team consisted of the principal investigator and two research assistants. To minimize information bias, standard operating procedures were established, and two research assistants received approximately 2 weeks of standardized training on study design, questionnaire content and research ethics prior to data collection. To reduce selection bias within the convenience sampling framework, the principal investigator screened all consecutive admissions during the study period by reviewing medical records to identify eligible patients. After providing informed consent, patients completed the questionnaires either on their own or with the help of a research assistant who read the questions aloud. To mitigate the burden on participants, demographic and clinical data were extracted directly from the hospital information system. As all participants were inpatients, they were asked to recall their SM behaviours at home during the previous treatment cycle. Completing the questionnaires took approximately 20 min. Research assistants performed on-the-spot checks to ensure data completeness, and any missing values were promptly rectified through direct confirmation with the participants.

### 2.6. Data Analysis

Data were analyzed using SPSS 25.0- and AMOS 26.0 (both IBM Corp., Armonk, NY, USA). Since data completeness was verified on-site during collection, no missing data imputation was required. Complete case analysis was performed for all 222 valid questionnaires. Assumptions of normality and absence of multicollinearity were examined prior to SEM analysis. Descriptive statistics were calculated to describe sociodemographic and clinical characteristics, IP, IS, and SM. Pearson’s correlation analysis was used to explore the relationships among the variables. Potential demographic and clinical confounders for NIP, PIP, IS, and SM were identified through a stepwise process involving univariate analysis followed by multivariable regression. Only statistically significant variables from the univariate analysis were retained for the following multivariable analysis. Consequently, performance status and educational level were recognized as covariates and were controlled for in the SEM (Supplementary Table S1).

We applied a two-phase data analysis procedure for the SEM, which included a measurement model and a structural model [49]. In the first step, a confirmatory factor analysis (CFA) was conducted to test the construct validity and reliability of the measurement model. Construct reliability and validity were evaluated using composite reliability (CR), and average variance extracted (AVE). Standardized factor loadings > 0.50, CR > 0.70, and AVE > 0.50 were considered acceptable [50].

In the second step, SEM was performed with AMOS 26.0 using maximum likelihood estimation to evaluate whether IS mediated the association between IP and SM. Direct, indirect, and total effects were estimated using 5000 bootstrap samples with 95% confidence intervals (CIs). Effects were considered statistically significant when the 95% CIs did not include zero [51]. Model fit for both the measurement and SEM was considered acceptable if the chi-square minimum/degrees of freedom (CMIN/DF) < 3.0, the root mean square error of approximation (RMSEA) < 0.08, the standardized root mean square residual (SRMR) < 0.08 and the confirmatory fit index (CFI), Tucker–Lewis Index (TLI), and Goodness of Fit Index (GFI) were all ≥0.90 [52].

## 3. Results

### 3.1. Sample Characteristics

In total, 229 questionnaires were handed out. Seven questionnaires were excluded because of invalid responses (e.g., logical inconsistencies or random response patterns), resulting in 222 valid questionnaires and an effective response rate of 96.94%. The mean age of participants was 68.43 years (SD = 5.76), with a male predominance. The majority of participants (77.03%) reported a per capita monthly household income of ≥4000 RMB. Regarding clinical characteristics, 39.19% had a disease duration exceeding one year, and 67.57% were diagnosed with adenocarcinoma (Table 1).

### 3.2. Descriptive Analysis of Study Variables

The mean score of SM was 3.80 ± 0.57. Of the subscales, daily life management scored the highest (3.99 ± 0.62), whereas symptom management demonstrated the lowest score (3.37 ± 0.64).

For PIP and NIP, relative to the midpoint of 3, all PIP dimensions, in addition to the NIP dimensions of consequence and timeline acute/chronic, showed a significantly elevated score (*p* < 0.05). Conversely, participants reported significantly lower levels of timeline cyclical and emotional representations compared to the midpoint (*p* < 0.05). Regarding the causal dimension, patients scored highest for environmental factors and lowest for previous illness or injury factors.

IS had a mean score of 4.86 ± 0.40, with firmness showing the highest mean score (5.14 ± 0.54) and creativity the lowest (3.86 ± 0.69) (Table 2).

### 3.3. Study Variable Correlations

Both SM and IS demonstrated a significant negative association with negative illness perception (NIP) (*r_SM_*: −0.500, −0.149, *p* < 0.05; *r_IS_*: −0.519, −0.250, *p* < 0.001), and a positive association with positive illness perception (PIP) (*r_SM_*: 0.360, 0.565, *p* < 0.001; *r_IS_*: 0.233, 0.467, *p* < 0.001). A positive relationship was observed between SM and IS (*r* = 0.427, *p* < 0.001). (Table 3).

### 3.4. SEM of Study Variables

Based on our theoretical framework and the observed correlations (Table 3), two SEM models were constructed to evaluate whether IS mediates the associations between IP (PIP and NIP) and SM. Multivariate normality was supported, with Mardia’s coefficients of 23.1 for the PIP model (*p* = 5; threshold = 35) and 32.4 for the NIP model (*p* = 6; threshold = 48) [53]. Additionally, following multicollinearity testing, diagnostics revealed a minimum tolerance value of 0.811 and a maximum variance inflation factor (VIF) of 1.233, indicating that no significant multicollinearity was present among the variables.

**Figure 2:**
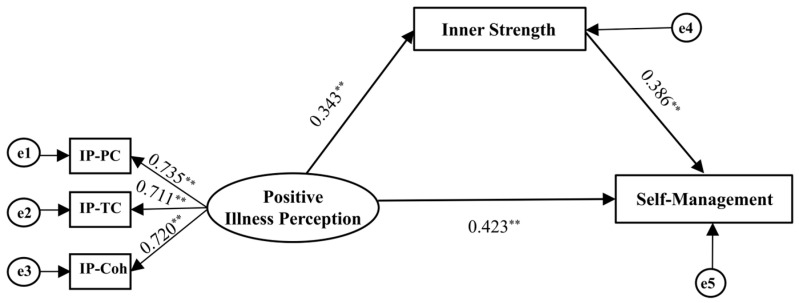
Structural equation model of inner strength as a mediator of positive illness perception and self-management among older adults with advanced lung cancer. Abbreviations: IP, illness perception; IP-PC, personal control; IP-TC, treatment control; IP-Coh, illness coherence. Note: ** *p* < 0.001; performance status and education level were controlled.

The structural model depicting the associations among PIP, IS, and SM is presented in Figure 2. Before testing the structural pathways, the measurement model was evaluated. Results indicated that standardized factor loadings ranged from 0.690 to 0.731, with a CR of 0.762 and an AVE of 0.511. The measurement model for PIP was saturated with zero degrees of freedom. Therefore, its adequacy was primarily established through standardized factor loadings, CR, and AVE, all of which met the recommended criteria. After adjusting for the covariates identified (performance status and educational level), the model fit the data well: CMIN/DF = 1.721, RMSEA = 0.045, SRMR = 0.033, CFI = 0.923, TLI = 0.937, and GFI = 0.914. SEM analysis revealed significant path coefficients from PIP to IS (*β* = 0.343, 95% CI: 0.193, 0.478; *p* < 0.001) and from IS to SM (*β* = 0.386, 95% CI: 0.216, 0.525; *p* < 0.001). The analysis further identified a significant indirect effect of PIP on SM via IS (*β* = 0.132, 95% CI: 0.011, 0.246; *p* = 0.028) alongside a direct effect (*β* = 0.423, 95% CI: 0.260, 0.563; *p* < 0.001). Thus, IS partially mediated the association between PIP and SM, and the mediation effect contributed 23.78% to the total effect (Table 4, Figure 2).

**Figure 3:**
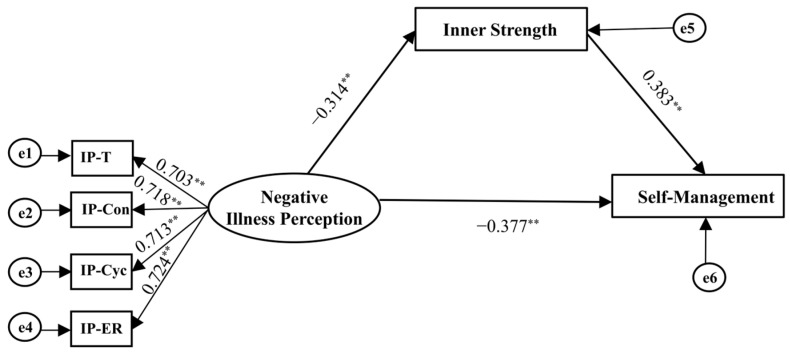
Structural equation model of inner strength as a mediator of negative illness perception and self-management among older adults with advanced lung cancer. Abbreviations: IP, illness perception; IP-T, timeline acute/chronic; IP-Con, consequence; IP-Cyc, timeline cyclical; IP-ER, emotional representations. Note: ** *p* < 0.001; performance status and education level were controlled.

The structural model of NIP and IS in predicting SM is presented in Figure 3. Before testing the structural pathways, the measurement model was evaluated. The results indicated that standardized factor loadings ranged from 0.692 to 0.726, with a CR of 0.767 and an AVE of 0.515. Additionally, the measurement model indicated a good fit (CMIN/DF = 1.931, RMSEA = 0.047, SRMR = 0.049, CFI =0.911, TLI = 0.915, and GFI = 0.908), fulfilling the prerequisites for subsequent structural pathway analysis. After adjusting for the covariates identified (performance status and educational level), the model fit the data well: CMIN/DF = 1.742, RMSEA = 0.048, SRMR = 0.036, CFI = 0.918, TLI = 0.931, and GFI = 0.911. SEM analysis revealed significant path coefficients from NIP to IS (*β* = −0.314, 95%CI: −0.453, −0.197; *p* < 0.001) and from IS to SM (*β* = 0.383, 95%CI: 0.220, 0.542; *p* < 0.001). The analysis further identified a significant indirect effect of NIP on SM via IS (*β*= −0.120, 95%CI: −0.234, −0.013; *p*= 0.032) alongside a direct effect (*β* = −0.377, 95%CI: −0.494, −0.221; *p* < 0.001). Thus, IS partially mediated the association between NIP and SM, and the mediation effect contributed 24.14% to the total effect (Table 4, Figure 3).

## 4. Discussion

To the best of our knowledge, this research is the first to investigate the associations among IP, IS, and SM in older adults with advanced lung cancer. Specifically, this study explored the mediating role of IS in the associations between IP (both PIP and NIP) and SM.

The overall SM score of older adults with advanced lung cancer in this study was higher than that reported by Du et al. [54]. This discrepancy could be attributed to the higher per capita monthly household income of our participants, who resided in an economically developed city. Higher income is often associated with easy access to better medical resources, which may facilitate the acquisition of SM-related knowledge and skills, potentially promoting more effective SM behaviors [35].

Notably, among the CSMAS subscales, symptom management had the lowest scores, significantly lower than those reported by Kong et al. [35]. Our findings highlight that symptom management in older adults with advanced lung cancer remains suboptimal, characterized by a marked deficiency in coping with treatment-related toxicities. Several critical factors may account for this inadequacy. First, lung cancer symptom clusters are inherently multifaceted and burdensome, posing substantially greater management challenges than other malignancies [55]. Second, despite the rapid paradigm shift toward targeted therapy and immunotherapy, a standardized clinical guidance framework has yet to be fully realized. Consequently, the efficacy of SM remains heavily contingent upon the professional expertise of individual healthcare providers [56]. Third, as this study focused on older adults, age-related cognitive decline and reduced learning capacity may pose significant barriers to mastering complex SM [57]. Furthermore, this study revealed that lower educational level, lower monthly household income per capita, and poorer performance status were associated with worse SM among patients. These findings suggest that older adults with advanced lung cancer have substantial supportive care needs in symptom management, and these needs deserve particular attention in clinical practice. In particular, patients with a lower educational level, lower monthly household income per capita, and poorer performance status should be regarded as priority targets for support. Therefore, healthcare professionals should develop individualized interventions based on patients’ specific conditions to strengthen their ability to recognize and cope with treatment-related toxicities. Helping patients identify core symptom clusters, initiate self-management for early warning symptoms, and receive multidimensional interventions, including medication, psychological support, and rehabilitation, may help reduce symptom burden and improve quality of life [28,58].

Regarding IP, we found that patients could identify an average of only 7.24 symptoms of lung cancer and had limited awareness of less common symptoms such as dysphagia, edema, and dizziness, consistent with the findings of Kahraman [59]. This may indicate that the supportive care needs of older adults with advanced lung cancer in terms of disease-related information and symptom recognition remain insufficiently addressed. Inadequate awareness of less common or atypical symptoms may delay patients in seeking help and hinder the timely initiation of appropriate SM. These findings highlight the importance of providing health education that is easy to understand and tailored to the cognitive characteristics of older adults throughout the disease trajectory in order to improve symptom recognition. PIP scores were significantly higher than the critical value of 3, indicating that patients had a relatively clear understanding of their disease and were more confident in disease control through treatment and management. However, this contradicts the conclusions of several previous studies [59,60,61]. This discrepancy may stem from two factors. First, the leading national status and high reputation of the hospital where we conducted the survey likely enhanced participants’ trust in treatment and PIP. Second, compared to earlier studies, the rapid advancement of lung cancer treatments in recent years has significantly improved prognoses, reinforcing patients’ hope and confidence in treatment. Furthermore, NIP scores (excluding timeline and consequence) were significantly below the critical value of 3, indicating that patients commonly perceived advanced lung cancer as a chronic disease prone to recurrence and with severe impacts on their QoL, consistent with prior research [59,60]. Interestingly, this study’s participants did not experience significant negative emotions, contrary to Kahraman and Pehlivan’s [59] findings but similar to the observations of Tufman et al. [62] for older adults with lung cancer and Stage IV lung cancer patients. Therefore, the discrepancy with Kahraman et al.’s [59] results may be due to our study population consisting of older adults. Our findings also suggest that older patients may report fewer negative emotions, possibly because accumulated life crises and illness experiences contribute to the development of higher IS, which may serve as a psychological buffer that helps them face illness-related challenges more positively [63].

In addition, this study identified several factors associated with IP. Patients with lower educational attainment and poorer physical functioning tended to report lower levels of PIP, whereas those with lower monthly household income per capita exhibited a weaker sense of personal control. These factors may jointly influence patients’ IP. One possible explanation is that lower educational attainment may limit patients’ ability to understand, integrate, and apply complex disease-related information, as well as reduce their access to relevant health information, thereby weakening PIP [64]. In contrast, poorer economic status may restrict access to high-quality medical resources and continuous care support, which may in turn undermine patients’ sense of control over disease management [65]. Meanwhile, patients with poorer physical functioning may be more likely to develop perceptions of the illness as uncontrollable because of activity limitations and a heavier disease burden [66]. These findings suggest that healthcare professionals should pay more attention to patients’ educational background, economic status, and physical functioning during clinical assessment in order to identify high-risk individuals at an early stage and provide targeted support.

The mean total score for IS in this study was 97.10 ± 7.86, which is similar to that reported by Viglund et al.’s [63] study of older adults, but higher than in Tan’s [67] study of patients with lung cancer who were receiving chemotherapy. This difference may be attributed to the fact that the patients in our study were older adults with advanced lung cancer. Although advanced lung cancer imposes a substantial physical and psychological burden, living with the illness over time may also contribute to the development of IS [63,68]. Furthermore, the study of Lundman et al. [69] showed that IS tends to increase with age, as older adults use life experience and wisdom to cope better. Among the subscales of ISS, creativity received the lowest score, which was also lower than that reported by Tan [67]. This finding may be related to the demographic characteristics of our sample, which consisted exclusively of older adults with advanced lung cancer. Previous research indicates that the creativity dimension exhibits significant age-related variability, with scores typically declining in older adults due to cognitive decline [70]. Moreover, this study found that patients with lower educational attainment and poorer physical functioning had lower levels of IS, suggesting that healthcare professionals should pay more attention to this subgroup. One possible explanation is that greater educational attainment may help patients better understand, integrate, and apply illness-related information, strengthen reflective coping, and more effectively mobilize internal psychological resources when facing adversity. Meanwhile, poorer physical functioning usually indicates a heavier symptom burden, greater activity limitations, and lower autonomy. This may not only weaken patients’ sense of control, but also reduce their opportunities to engage in social interaction and meaningful activities, thereby further undermining their IS. These findings further suggest that supportive care for older adults with advanced lung cancer should not be limited to symptom management and symptom recognition, but should also give full consideration to their psychosocial needs. By helping patients maintain emotional connections with family and friends, encouraging engagement in meaningful activities, and valuing their ideas and suggestions for coping with treatment-related problems, healthcare professionals may help strengthen patients’ IS and thereby support better disease management [24].

This study further supports the association between IP and SM. Specifically, PIP was positively correlated with SM, while NIP was negatively correlated with SM, similar to the findings of studies on other patients with cancer [29,30]. A plausible explanation is that patients with a strong sense of personal control over their disease tend to be more proactive in facing challenges, more motivated to acquire the knowledge and skills needed for SM, and more likely to adopt positive SM [71,72]. Conversely, patients who regard their illness as more threatening and severe often experience emotional distress, which may deplete their confidence and impede their SM [26]. Therefore, healthcare professionals should focus on the influence of disease-related perceptions on older adults with advanced lung cancer and provide support to enhance their SM.

Similar to findings in a study of female cancer patients, IS was positively associated with SM among older adults with advanced lung cancer [40]. A qualitative study of older adults highlighted that IS is expressed by actively engaging in treatment, making decisions in collaboration with clinicians, and proactively seeking health information to reduce anxiety [43]. Consequently, among older adults with advanced lung cancer, those with higher IS may be more likely to engage in effective SM.

Regarding the SEM results, this study offers initial evidence for the mediating role of IS and suggests a potential pathway linking IP to SM among older adults with advanced lung cancer. This finding is broadly consistent with Stress and Coping Theory [39], which posits that individuals cope with illness-related stress through primary appraisal of the threat and secondary appraisal of available coping resources. Within this theoretical framework, IP may reflect how patients appraise the threat and significance of advanced lung cancer, whereas IS may represent an important psychological resource mobilized during the coping process. From this perspective, IS may function as a psychological buffer through which negative illness-related perceptions are transformed into more positive SM behaviors. More specifically, our findings suggest that both PIP and NIP may influence SM partially through IS. Patients who perceive their illness as more understandable and controllable may be more likely to mobilize inner psychological resources, respond more positively to illness-related challenges, and maintain engagement in SM. In contrast, older adults with advanced lung cancer who have higher levels of NIP may be more likely to experience a gradual depletion of IS owing to persistent symptom burden, prognostic uncertainty, and emotional distress [73], which may in turn substantially impair their motivation and behaviors related to SM. For the vulnerable population of older adults with advanced lung cancer, clarification of this mediating mechanism may have important clinical implications. From a clinical perspective, these findings highlight the importance of closely monitoring both IP and IS when supporting SM among older adults with advanced lung cancer. In particular, patients who lack confidence in disease control or perceive the illness as an overwhelming threat may require additional psychosocial support. Professional interventions such as cognitive-behavioral therapy, mindfulness-based interventions, and empathic communication may help patients attenuate NIP, reconstruct their sense of mastery in disease management, and thereby effectively activate their inner psychological resources [74]. Additional effective strategies for enhancing IS and promoting SM may include helping patients to maintain close emotional connections with family members and friends, as well as encouraging participation in meaningful daily activities [24].

## 5. Strengths and Limitations

As far as we know, this study is one of the few to focus specifically on the vulnerable population of older adults with advanced lung cancer. By utilizing SEM to examine the mediating role of IS as an important psychological resource between IP and SM, this study not only provides empirical support for the applicability of Stress and Coping Theory in this population but also presents a preliminary empirical basis for developing strategies that target psychosocial intervention.

Despite its strengths, this study has several limitations that should be considered. First, this study employed a cross-sectional design, which limits our ability to infer causal relationships among variables. Although the SEM demonstrated statistical pathways from IP to SM via the mediation of IS, bidirectional relationships may exist in reality. For example, successful experiences in SM could also enhance a patient’s IS and PIP. Consequently, the mediation mechanisms proposed in our study should be perceived as exploratory, and future longitudinal research is warranted to confirm these causal pathways. Second, relying on convenience sampling from a single medical center in Guangzhou, China, inevitably introduced selection bias. Patients who were willing and physically able to complete the questionnaires might represent a group with higher PIP, greater levels of IS, or more proactive SM behaviors from the outset. Therefore, one should be cautious when generalizing these findings to older adults with advanced lung cancer in other socioeconomic or cultural contexts. Finally, relying on self-reported data, despite using validated scales, may have introduced recollection bias or socially desirable responding. Future studies should adopt objective behavioral indicators or observational data for a more multidimensional assessment.

## 6. Conclusions

The study’s results highlight that, among older adults with advanced lung cancer, PIP and IS were positively associated with SM, whereas NIP was negatively associated with SM. Notably, IS may partially mediate the association between IP and SM. These findings have important clinical implications. As SM levels in older adults with advanced lung cancer remain suboptimal, clinicians should prioritize the assessment of IP to facilitate targeted interventions that improve disease understanding and confidence in disease control, while mitigating negative emotional responses. Additionally, healthcare professionals are encouraged to cultivate patients’ IS by fostering social participation, thereby mobilizing patients’ internal resources essential for effective disease SM.

## Figures and Tables

**Table 1:** Demographic and clinical variables (*N* = 222).

Variables	*n*	%
Sex		
Male	143	64.41
Female	79	35.59
Age (years)		
60–69	135	60.81
≥70	87	39.19
Educational level		
Primary school or below	70	31.53
Middle school	70	31.53
High school	39	17.57
College or above	43	19.37
Per capita monthly household income (RMB)		
<4000	51	22.97
4000–5999	83	37.39
6000–7999	63	28.38
≥8000	25	11.26
Histological classification		
Adenocarcinoma	150	67.57
Squamous cell carcinoma	34	15.32
Neuroendocrine carcinoma	32	14.41
Other ^1^	6	2.70
Tumor stage		
III	46	20.72
IV	176	79.28
Disease duration (years)		
≤0.5	83	37.39
0.6–0.9	52	23.42
1–2.9	56	25.23
≥3	31	13.96
Performance status ^2^		
0	62	27.93
1	137	61.71
2	23	10.36

Note: ^1^ Comprises adenosquamous carcinoma (*n* = 1), sarcomatoid carcinoma (*n* = 1), and large cell carcinoma (*n* = 4). ^2^ Performance status was assessed using the Eastern Cooperative Oncology Group (ECOG) scale, ranging from 0 (fully active) to 5 (deceased) [45]. Patients with a performance status ≥3 were excluded from this study [45].

**Table 2:** Means and standard deviations for self-management, illness perception, and inner strength (*N* = 222).

Variable	Subscales/Factors	Mean	SD
SM	Total self-management	3.80	0.57
	Daily life management	3.99	0.62
	Symptom management	3.37	0.64
	Emotional management	3.97	0.55
	Communication with healthcare providers	3.61	0.88
	Information management	3.95	0.68
IP			
Identity		7.21	2.04
Cause	Environmental factors	2.12	0.82
	Behavioral factors	2.03	0.87
	Immunological factors	1.91	0.99
	Psychological factors	1.79	0.83
	Previous illness or injury factors	1.35	0.55
PIP	Personal control	3.65	0.62
	Treatment control	3.66	0.57
	Illness coherence	3.56	0.66
NIP	Consequence	3.39	0.62
	Timeline cyclical	2.78	0.69
	Timeline acute/chronic	3.81	0.52
	Emotional representations	2.30	0.53
IS	Total inner strength	4.86	0.40
	Firmness	5.14	0.54
	Creativity	3.86	0.69
	Courage	4.59	0.62
	Connectedness	5.10	0.67
	Flexibility	5.13	0.49

Abbreviations: SD, standard deviation; PIP, positive illness perception; NIP, negative illness perception; IS, inner strength; SM, self-management.

**Table 3:** Relationships between self-management, illness perception, and inner strength (*N* = 222).

Variable	Subscales/Factors	SM	IS
IP		Total score	Total score
Identity		0.103	0.041
Cause	Environmental factors	0.108	0.043
	Behavioral factors	0.039	0.092
	Immunological factors	0.027	0.043
	Psychological factors	0.001	0.074
	Previous illness or injury factors	0.037	0.067
PIP	Personal control	0.565 **	0.467 **
	Treatment control	0.360 **	0.405 **
	Coherence	0.457 **	0.233 **
NIP	Consequence	−0.385 **	−0.304 **
	Timeline cyclical	−0.330 **	−0.250 **
	Timeline acute/chronic	−0.149 *	−0.276 **
	Emotional representations	−0.500 **	−0.519 **
IS	Total score	0.427 **	

Abbreviations: PIP, positive illness perception; NIP, negative illness perception; IS, inner strength; SM, self-management. Note: * *p* < 0.05, ** *p* < 0.001.

**Table 4:** Predictive effects of illness perception and inner strength on self-management (*N* = 222).

Structural Paths	Estimate	95% CIs		Relative Effect Value (%)
		Lower	Upper	
Predictive path via PIP				
PIP→IS	0.343 **	0.193	0.478	
IS→SM	0.386 **	0.216	0.525	
Direct effect: PIP→SM	0.423 **	0.260	0.563	76.22
Indirect effect: PIP→IS→SM	0.132 *	0.011	0.246	23.78
Predictive path via NIP				
NIP→IS	−0.314 **	−0.453	−0.197	
IS→SM	0.383 **	0.220	0.542	
Direct effect: NIP→SM	−0.377 **	−0.494	−0.221	75.86
Indirect effect: NIP→IS→SM	−0.120 *	−0.234	−0.013	24.14

Abbreviations: PIP, positive illness perception; NIP, negative illness perception; IS, inner strength; SM, self-management. * *p* < 0.05, ** *p* < 0.001.

## Data Availability

The datasets presented in this article are not readily available because the data are part of an ongoing study. Requests to access the datasets should be directed to the corresponding author.

## Supplementary Materials

The following supporting information can be downloaded at https://www.mdpi.com/article/10.3390/healthcare14101257/s1: Table S1: Results of univariate and multivariate analyses for identifying candidate covariates (*N* = 222).

## Abbreviations

The following abbreviations are used in this manuscript:

SM	Self-management
CSM	Common-Sense Model of Self-Regulation
IP	Illness perception
NIP	Negative illness perception
PIP	Positive illness perception
IS	Inner strength
STROBE	Strengthening the Reporting of Observational Studies in Epidemiology
CSMAS	Cancer Self-Management Assessment Scale
IPQ-R	Revised Illness Perception Questionnaire
ISS	Inner Strength Scale
GFI	Goodness of fit index
SD	Standard deviation
CI	Confidence interval
