# Supplementary material for: The Mediating Role of Inner Strength in the Relationship Between Illness Perception and Self-Management in Older Adults with Advanced Lung Cancer: A Cross-Sectional Study
Source: Healthcare (Basel). 2026 May 7;14(10):1257. doi: 10.3390/healthcare14101257 (PMC13205715; doi:10.3390/healthcare14101257)
Supplement: Supplementary file 1 [file healthcare-14-01257-s001.zip › healthcare-4157478-supplementary.pdf]

Supplementary Material

Supplementary Table S1. Results of univariate and multivariate analyses for identifying candidate covariates *(N = 222)*

Outcome variable	Candidate covariates	Univariate test (*P*-value)	Multivariate *β* (P-value)	Consideration for control
PIP-Personal control	Sex	0.443	0.102 (0.133)	Not retained
	Age	0.517	0.031 (0.630)	Not retained
	Educational level	$<0.05^*$	0.242 ($<0.05^*$)	Retained
	Per capita monthly household income	$<0.05^*$	0.236 ($<0.05^*$)	Dimension-specific significance
	Histological classification	$<0.05^*$	−0.035 (0.613)	Not retained
	Tumor staging	$<0.05^*$	0.070 (0.321)	Not retained
	Disease duration (years)	0.452	−0.076 (0.306)	Not retained
	Performance status	$<0.05^*$	−0.581 ($<0.05^*$)	Retained
PIP-Treatment control	Sex	0.473	0.013 (0.853)	Not retained
	Age	$<0.05^*$	0.038 (0.560)	Not retained
	Educational level	$<0.05^*$	0.231 ($<0.05^*$)	Retained
	Per capita monthly household income	0.274	0.061 (0.107)	Not retained
	Histological classification	0.463	−0.02 (0.542)	Not retained
	Tumor staging	0.361	−0.013 (0.766)	Not retained
	Disease duration (years)	0.367	0.009 (0.631)	Not retained
	Performance status	$<0.05^*$	−0.251 ($<0.05^*$)	Retained
PIP-Illness coherence	Sex	0.473	−0.016 (0.851)	Not retained
	Age	0.324	0.005 (0.948)	Not retained
	Educational level	$<0.05^*$	0.218 ($<0.05^*$)	Retained
	Per capita monthly household income	0.574	0.024 (0.548)	Not retained
	Histological classification	0.463	−0.031 (0.384)	Not retained
	Tumor staging	0.461	−0.034 (0.449)	Not retained
	Disease duration (years)	0.323	0.034 (0.105)	Not retained
	Performance status	$<0.05^*$	−0.243 ($<0.05^*$)	Retained
NIP-Consequence	Sex	0.382	0.062 (0.486)	Not retained

	Age	0.374	−0.230 (<0.05*)	Dimension-specific significance
	Educational level	<0.05*	−0.227 (<0.05*)	Retained
	Per capita monthly household income	0.293	−0.064 (0.121)	Not retained
	Histological classification	0.463	0.001 (0.969)	Not retained
	Tumor staging	0.521	−0.063 (0.173)	Not retained
	Disease duration (years)	0.387	0.003 (0.878)	Not retained
	Performance status	<0.05*	−0.248 (<0.05*)	Retained
NIP-Timeline cyclical	Sex	0.371	−0.023 (0.808)	Not retained
	Age	0.393	−0.119 (0.142)	Not retained
	Educational level	<0.05*	−0.278 (<0.05*)	Retained
	Per capita monthly household income	0.394	−0.066 (0.137)	Not retained
	Histological classification	0.483	−0.055 (0.169)	Not retained
	Tumor staging	<0.05*	0.176 (<0.05*)	Dimension-specific significance
	Disease duration (years)	0.497	0.045 (0.051)	Not retained
	Performance status	<0.05*	0.295 (<0.05*)	Retained
NIP-Timeline acute/chronic	Sex	0.443	−0.023 (0.757)	Not retained
	Age	0.297	0.047 (0.461)	Not retained
	Educational level	<0.05*	0.226 (<0.05*)	Retained
	Per capita monthly household income	0.394	−0.016 (0.639)	Not retained
	Histological classification	0.524	−0.014 (0.645)	Not retained
	Tumor staging	0.632	0.069 (0.081)	Not retained
	Disease duration (years)	0.282	0.023 (0.196)	Not retained
	Performance status	<0.05*	−0.218 (<0.05*)	Retained
NIP-Emotional representations	Sex	0.251	0.100 (0.186)	Not retained
	Age	<0.05*	−0.155 (<0.05*)	Dimension-specific significance
	Educational level	<0.05*	−0.248 (<0.05*)	Retained
	Per capita monthly household income	<0.05*	−0.172 (<0.05*)	Dimension-specific significance
	Histological classification	0.244	0.026 (0.403)	Not retained
	Tumor staging	<0.05*	−0.143（0.631）	Not retained
	Disease duration (years)	0.052	−0.017 (0.338)	Not retained
	Performance status	<0.05*	0.149 (<0.05*)	Retained
IS	Sex	0.143	0.007(0.915)	Not retained
	Age	<0.05*	−0.002 (0.976)	Not retained
	Educational level	<0.05*	0.293 (<0.05*)	Retained

	Per capita monthly household income	0.246	0.120 (0.083)	Not retained
	Histological classification	0.364	−0.044 (0.536)	Not retained
	Tumor staging	0.311	−0.026 (0.711)	Not retained
	Disease duration (years)	0.082	0.02 (0.769)	Not retained
	Performance status	＜0.05*	−0.286 (＜0.05*)	Retained
SM	Sex	0.143	−0.041 (0.505)	Not retained
	Age	0.317	−0.022 (0.715)	Not retained
	Educational level	＜0.05*	0.325 (＜0.05*)	Retained
	Per capita monthly household income	＜0.05*	0.232 (＜0.05*)	Dimension-specific significance
	Histological classification	0.364	−0.012 (0.385)	Not retained
	Tumor staging	0.882	0.056 (0.385)	Not retained
	Disease duration (years)	0.052	0.067 (0.264)	Not retained
	Performance status	＜0.05*	−0.197 (＜0.05*)	Retained

Abbreviations: PIP, Positive illness perception, NIP, Negative illness perception, IS, Inner strength, SM, Self-management

Note: $^*p<0.05$,
